# Lichen myxedematosus associated with monoclonal gammopathy of undetermined significance: A case report and literature review

**DOI:** 10.3389/fmed.2023.1118555

**Published:** 2023-03-08

**Authors:** Hua Huang, Shen-Xian Qian

**Affiliations:** Department of Hematology, Affiliated Hangzhou First People's Hospital, Zhejiang University School of Medicine, Hangzhou, China

**Keywords:** monoclonal gammopathy, monoclonal gammopathy of clinical significance, monoclonal gammopathy and skin changes syndrome, thalidomide, glucocorticoids, lichen myxedematosus

## Abstract

Lichen myxedematosus (LM) is an idiopathic cutaneous mucinosis disorder, and monoclonal gammopathy of undetermined significance (MGUS) is a preneoplastic plasma cell disease with a monoclonal increase in globulin. Patients with LM combined with monoclonal gammopathy are normally diagnosed with scleromyxedema. However, we report a case of generalized papules combined with MGUS in a 78-year-old man who was eventually diagnosed with atypical or intermediate forms of LM because it only involved the skin, and the pathological type was not consistent with scleromyxedema. Few cases of atypical or intermediate forms of LM have been reported, so the course of atypical or intermediate forms of LM is unpredictable. We report the diagnosis and treatment of a case of atypical forms of LM to discuss the current understanding of the disease, hoping to provide a reference for clinical research on this disease.

## Introduction

Lichen myxedematosus (LM) is an idiopathic cutaneous mucinosis disorder characterized by lichenoid papules due to mucin dermal deposition without thyroid dysfunction. It was classified by Rongioletti in 2001 into three distinct clinicopathological subsets: (i) Scleromyxedema: Extensive and sclerodermoid LM associated with monoclonal gammopathy and systemic manifestations; (ii) Localized forms: LM in the absence of monoclonal gammopathy and systemic involvement; and (iii) Atypical or intermediate forms: LM not meeting the criteria of either scleromyxedema or the localized form (1).

Monoclonal gammopathy of undetermined significance (MGUS) is a preneoplastic plasma cell disease with a monoclonal increase in globulin, which is characterized by a serum monoclonal protein level of less than 30 g/L, a percent of bone marrow clonal plasma cells of less than 10%, and the absence of plasma cell myeloma-associated end-organ damage (hypercalcemia, renal insufficiency, anemia, or bone injury). There were no B-cell lymphomas or other diseases known to produce monoclonal protein as well (2).

We report a case of a patient with generalized papules associated with MGUS who was diagnosed with atypical or intermediate forms of LM after a complete examination.

## Case presentation

### Chief complaints

The patient was a 78-year-old man who had generalized brown rashes with pruritus for more than 40 years.

### History of present illness

Forty years previously, the patient had scattered red papules on the trunk without pruritus, which gradually increased and spread to the skin, such as the limbs and buttocks. Severe pruritus began to appear, and the papules turned brown in color.

### History of past illness

The patient had coronary heart disease for more than 10 years, atrial fibrillation for 1 year, mitral valve artificial valve replacement and pacemaker implantation, and type 2 diabetes for 2 years.

### Personal and family history

The patient denied any family history of similar diseases.

### Physical examination

**Figure 1:**
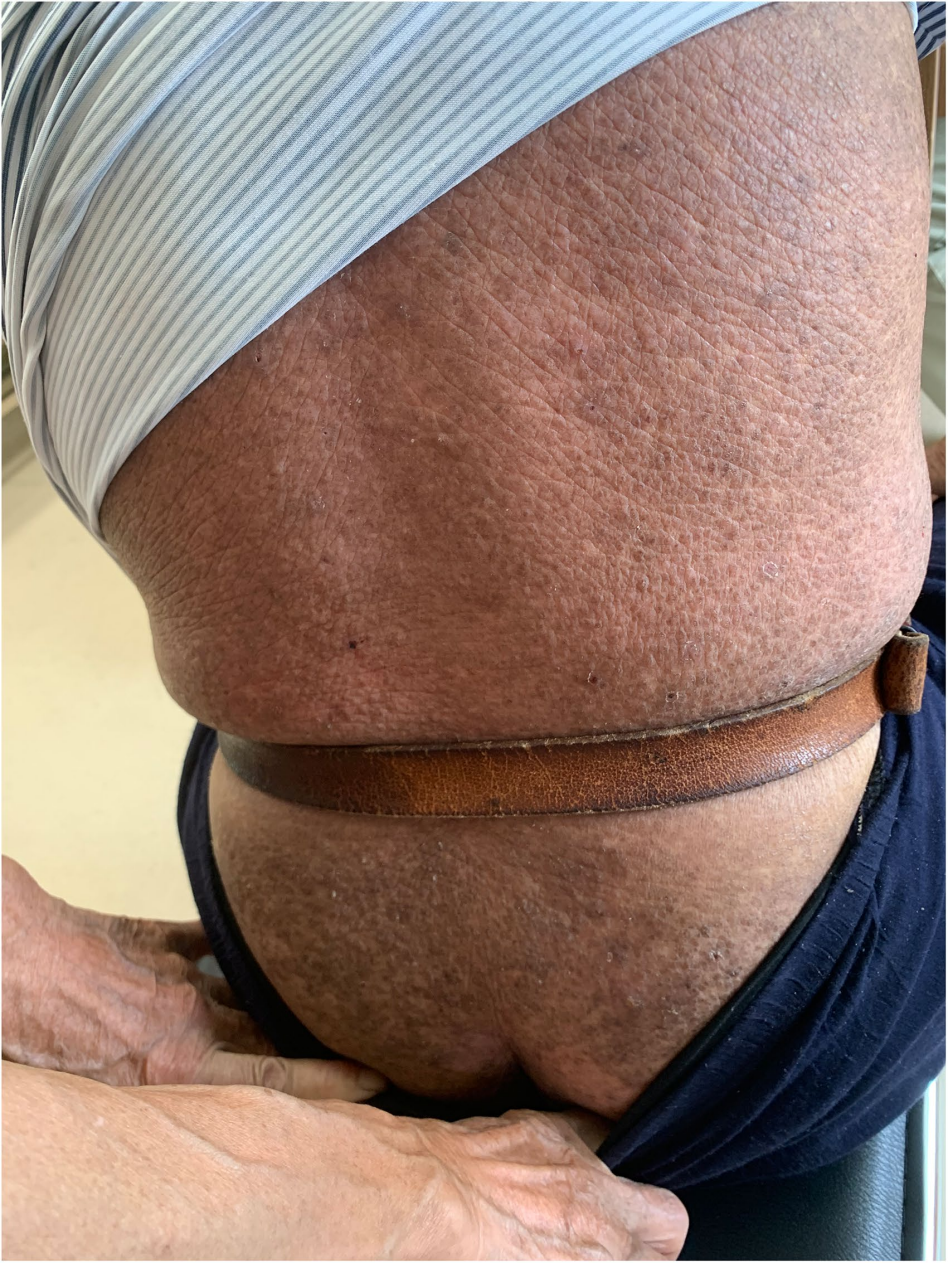
Cutaneous lesions on the trunk and buttocks.

**Figure 2:**
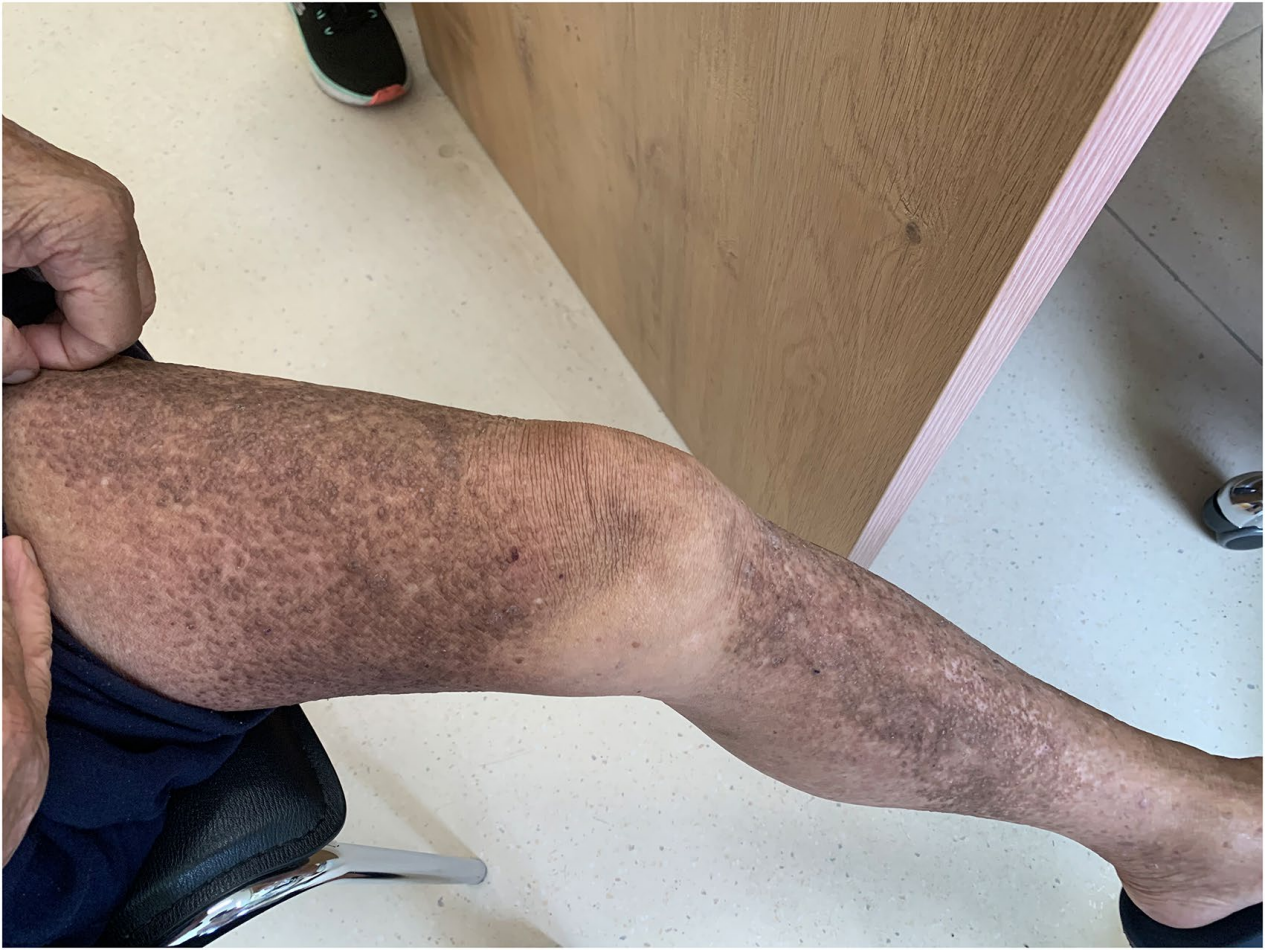
Cutaneous lesions on the thigh.

On physical examination, the vital signs were as follows: body temperature, 36.4°C; blood pressure, 124/71 mmHg; heart rate, 62 beats per min; and respiratory rate, 18 breaths per min. Furthermore, there were extensive, dense, waxy, dark brown, dome-shaped papules measuring 2–3 mm on the skin of the extremities, trunk, and buttocks in a symmetric pattern (Figures 1, 2).

### Laboratory examinations

After admission, blood IgG was 18.1 g/L (normal<16.9 g/L), and K light chain was 17.7 g/L (normal<13.5 g/L). There were no significant abnormalities in the other parameters, such as body weight, routine blood, liver function, kidney function, hepatitis virus and HIV DNA tests, blood glucose and blood fat.

### Imaging examinations

No obvious amyloid changes were found on cardiac ultrasound. Chest CT revealed no abnormalities.

## Further diagnostic work-up

**Figure 3:**
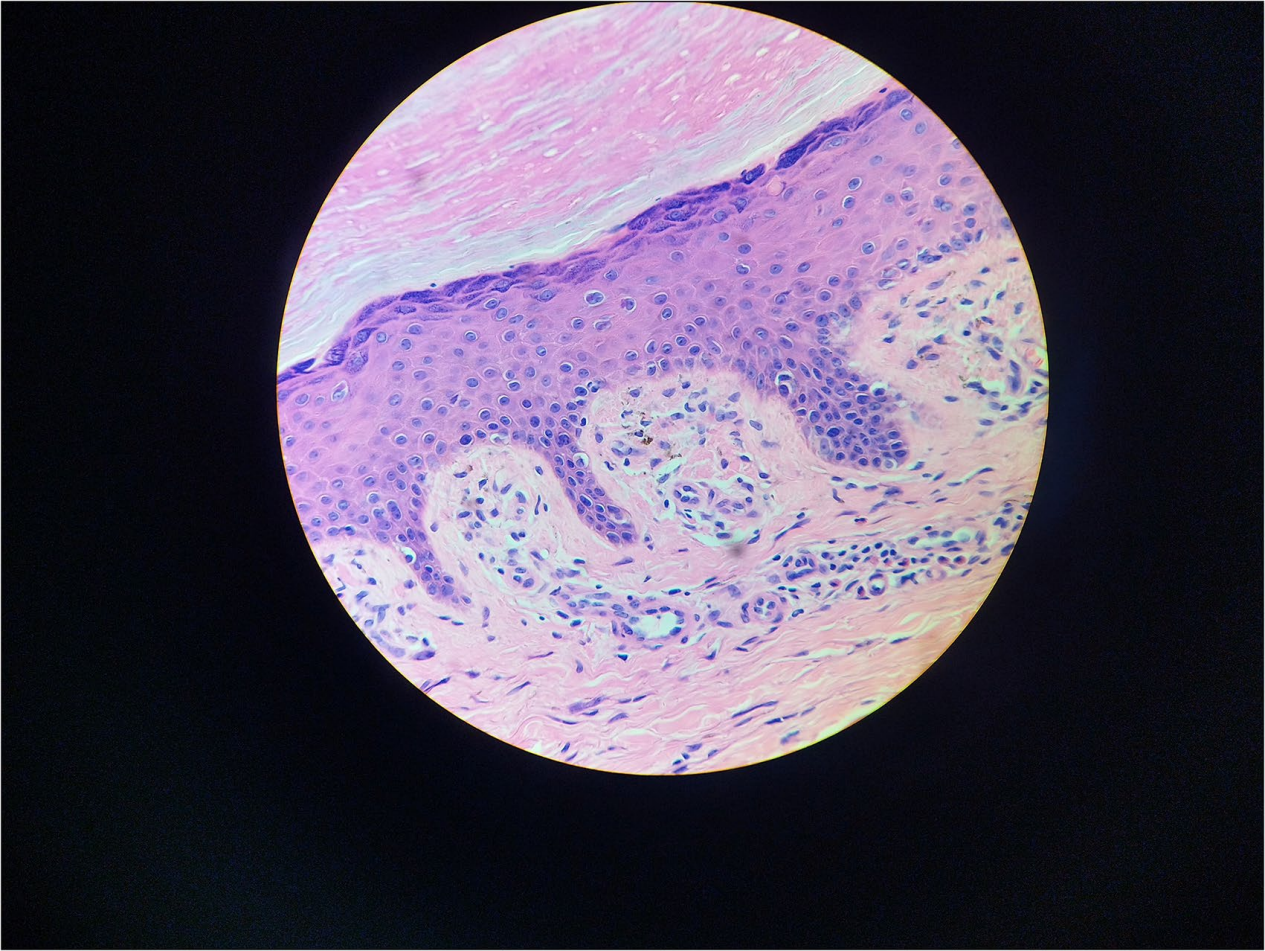
Hematoxylin–eosin stain 400X.

**Figure 4:**
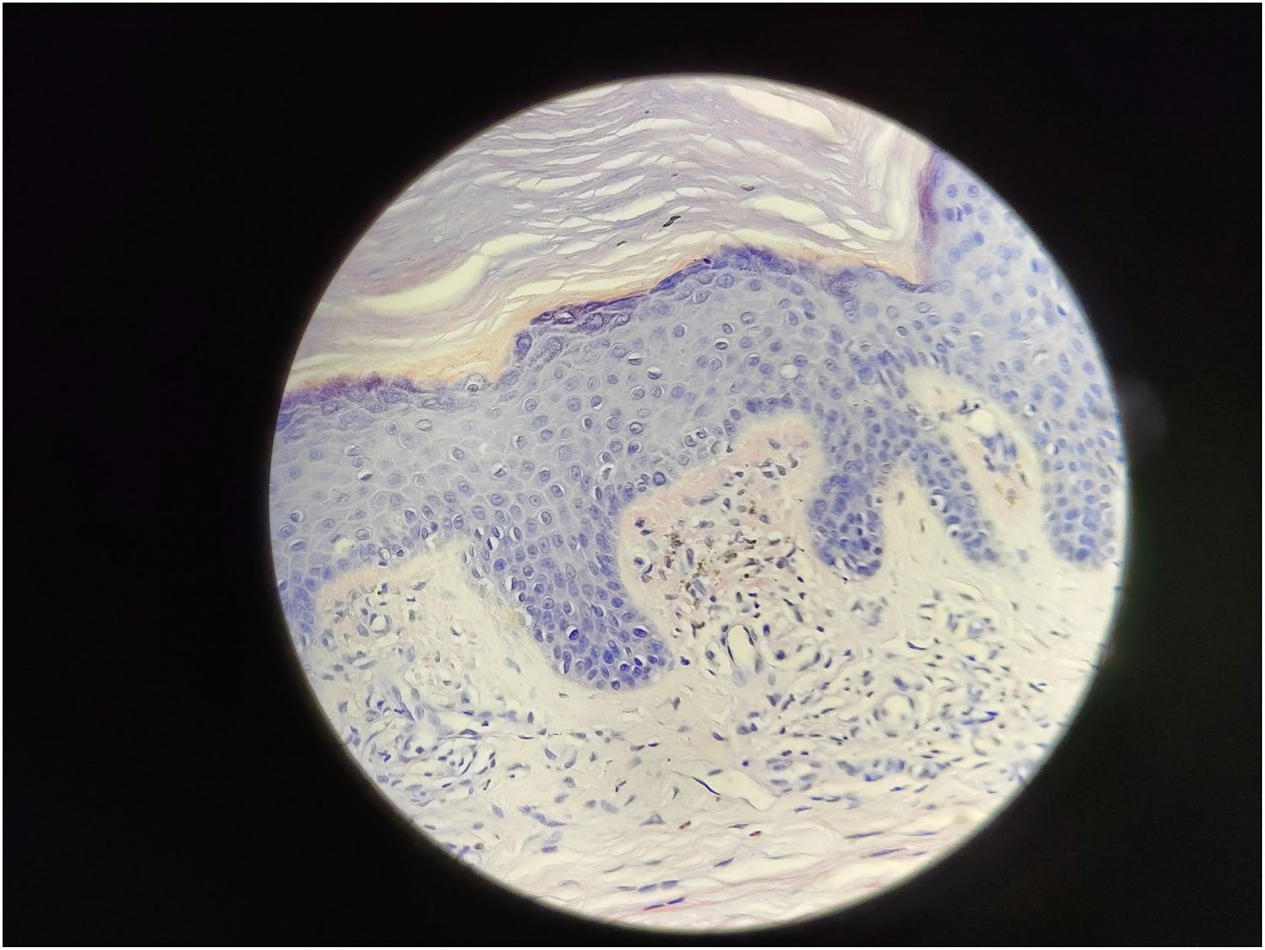
Congo red stain 400x.

Serum immunofixation electrophoresis showed that the monoclonal protein was of the IgG-K type. Bone marrow examination, bone marrow biopsy, bone marrow flow cytometry, and bone marrow karyotype analysis showed no abnormalities. The patient was diagnosed with MGUS. There was a high suspicion of scleromyxedema, and a decision was made to perform a skin biopsy. A small piece of skin tissue in the abdominal wall showed hyperplasia of the surface stratum corneum, a reddish mass in the papillary dermis, positive staining for Congo red, proliferation and dilation of capillaries in the focal papillary layer, pigment incontinence, and focal infiltration of lymphocytes and eosinophils around the blood vessels in the dermal layer. Special staining: Congo red staining was positive, and Masson staining was positive (Figures 3, 4).

There were no obvious abnormalities in autoimmune antibodies, thyroid function, or renal function. According to the detailed medical history inquiry and physical examination, it was found that the patient had no obvious involvement of other systems except the skin.

## Final diagnosis

After discussion and literature review, the patient was diagnosed with atypical forms of LM.

## Treatment

After dermatological consultation, the patient was treated with oral glucocorticoids combined with ointment for a month, to which there was no satisfactory response, and the skin lesions and pruritus persisted. Therefore, considering the potential relationship between MGUS and LM in this case, the patient was treated with thalidomide 100 mg once a day and dexamethasone 3.75 mg twice a day.

## Outcome and follow-up

After 2 weeks of glucocorticoid combined with thalidomide treatment, the rash has not changed significantly, but the patient reports that the pruritus is better than before. It may be that the drugs are still taking time to show their effects, and the patient is still in follow-up.

## Discussion

LM is an idiopathic cutaneous mucinosis disorder characterized by lichenoid papules due to mucin dermal deposition without thyroid dysfunction. It was classified by Rongioletti (1) in 2001 into three distinct clinicopathological subsets: (i) scleromyxedema; (ii) localized forms, and (iii) atypical or intermediate forms. This third subtype, atypical or intermediate forms, includes those LMs that do not meet the first two diagnostic criteria, such as scleromyxedema without monoclonal gammopathy, localized forms with monoclonal gammopathy and/or systemic symptoms, localized forms with a mixture of multiple subtypes, and other unspecified conditions. In 2017, Nofal et al. (3) suggested that LM should be classified into systemic and pure cutaneous types according to the presence or absence of systemic symptoms to avoid the previous confusion of the third category, atypical or intermediate forms. However, this classification needs to be supported by more research.

Because of the generalized papules and MGUS in this case, the first clinical consideration was scleromyxedema, a subtype of LM, which often has systemic involvement and is even life-threatening. Scleromyxedema is also considered to be a form of monoclonal gammopathy of cutaneous significance because of its high association (>50%) with monoclonal globulin (4, 5). However, the histological findings did not support the diagnosis of scleromyxedema. The microscopic triad, composed of mucin deposition (composed primarily of hyaluronic acid) in the upper and mid-reticular dermis, fibrosis and irregularly arranged fibroblast proliferation, was not found (1). The microscopic pictures showed that mucin deposition was limited to the upper (papillary) dermis, and there was no fibrosis or fibroblast proliferation. Finally, the patient was diagnosed with atypical forms of LM.

There is no need for treatment for localized forms of LM, and a wait-and-see approach is recommended (1, 6–8). In terms of treatment for scleromyxedema, intravenous immunoglobulin, systemic glucocorticoids, thalidomide, autologous stem cell transplantation and other therapies have been reported (9). Because so few cases have been reported, the course of atypical or intermediate forms of LM is unpredictable, and therefore, such cases should be followed up carefully.

Considering the potential relationship between papules and pruritus and MGUS in this case, thalidomide combination therapy was added when glucocorticoids alone were ineffective. Thalidomide is able to obtain a significant and long-lasting response with significantly reduced paraprotein levels. It also has well-known antiangiogenic, anti-inflammatory, and immunomodulatory properties (10).

There are still many unknowns in the pathogenesis, pathophysiology, treatment regimen and other aspects of LM associated with MGUS. It is hoped that more studies will be conducted to promote the understanding of LM associated with MGUS in the future.

## Conclusion

This is a case of LM associated with MGUS treated with a combination of glucocorticoids and thalidomide. After 2 weeks of glucocorticoids combined with thalidomide treatment, the patient’s pruritus improved, but the papules did not change significantly. There is still little clinical awareness of LM associated with MGUS, and patients with this disease may still suffer from prolonged pain. On the one hand, careful follow-up is necessary to monitor disease progression. On the other hand, we should also actively administer symptomatic treatment to improve the quality of life of patients. More research needs to be done.

## Data availability statement

The original contributions presented in the study are included in the article/supplementary material, further inquiries can be directed to the corresponding author.

## Ethics statement

Written informed consent was obtained from the individual(s) for the publication of any potentially identifiable images or data included in this article.

## Author contributions

HH reviewed the literature and wrote the first and final drafts of the manuscript. S-XQ is responsible for the full text guidance and communication. All authors contributed to the article and approved the submitted version.

## Conflict of interest

The authors declare that the research was conducted in the absence of any commercial or financial relationships that could be construed as a potential conflict of interest.

## Publisher’s note

All claims expressed in this article are solely those of the authors and do not necessarily represent those of their affiliated organizations, or those of the publisher, the editors and the reviewers. Any product that may be evaluated in this article, or claim that may be made by its manufacturer, is not guaranteed or endorsed by the publisher.
